# Meta-analysis of detection of respiratory events during procedural sedation

**DOI:** 10.1186/cc9770

**Published:** 2011-03-11

**Authors:** JB Waugh, YA Khodneva, CA Epps

**Affiliations:** 1 University of Alabama at Birmingham, AL, USA

## Introduction

The use of procedural sedation and analgesia (PSA) has increased in frequency and scope, including emergent settings inside and outside the hospital. Although end-tidal CO_2 _(EtCO_2_) monitoring is routinely used during general anesthesia to monitor ventilatory status, this is not the case for PSA. Pulse oximetry and visual inspection, both with inherent limitations, represent the current standards of care for monitoring ventilatory status during PSA. EtCO_2 _monitoring may be a preferable method for detecting alveolar hypoventilation and preventing hypoxemia during PSA but is not widely used in this setting. Our study objective was to determine whether capnography in addition to standard monitoring improved detection of respiratory events compared with standard monitoring alone.

## Methods

A literature search was conducted using the electronic databases PubMed, CINAHL, and Cochrane Library (Cochrane Reviews, CENTRAL) for studies published between 1995 and 2009 reporting adverse respiratory events during procedural sedation and analgesia with clearly defined EtCO_2 _threshold, clear study design, *P-*value calculation, similar outcome and predictor variable definitions, and binary independent and dependent variable raw data. To limit threats from variations in practice, only reports of adults in the USA were included. Five such studies were evaluated independently. A meta-analysis of these studies was performed.

## Results

During PSA, cases of respiratory depression were 17.6 times more likely to be detected if monitored by capnography, versus cases not monitored by capnography (95% CI, 2.5 to 122.1; *P *< 0.004).

## Conclusions

This analysis suggests that EtCO_2 _monitoring is an important addition for detecting respiratory depression during PSA.

